# RETRACTION: Role of TGF‐β3 in Modulating Inflammatory Responses and Wound Healing Processes in Ischemic Ulcers in Atherosclerotic Patients

**DOI:** 10.1111/iwj.70537

**Published:** 2025-04-18

## Abstract

**RETRACTION:** MaT. Y. , TangS. L. , WangB. , WangG. , SunC. M. , PanJ. X. , HanD. Q. , LiJ. Y. , and ZhongJ. H. , “Role of TGF‐β3 in Modulating Inflammatory Responses and Wound Healing Processes in Ischemic Ulcers in Atherosclerotic Patients,” International Wound Journal 21, no. 2 (2024): e14762, 10.1111/iwj.14762.38356162 PMC10867290

The above article, published online on 14 February 2024, in Wiley Online Library (http://onlinelibrary.wiley.com/), has been retracted by agreement between the journal Editor in Chief, Professor Keith Harding; and John Wiley & Sons Ltd. Following an investigation by the publisher, all parties have concluded that this article was accepted solely on the basis of a compromised peer review process. The editors have therefore decided to retract the article. The authors did not respond to our notice regarding the retraction.

**RETRACTION:** T. Y. Ma , S. L. Tang , B. Wang , G. Wang , C. M. Sun , J. X. Pan , D. Q. Han , J. Y. Li , and J. H. Zhong , “Role of TGF‐β3 in Modulating Inflammatory Responses and Wound Healing Processes in Ischemic Ulcers in Atherosclerotic Patients,” International Wound Journal 21, no. 2 (2024): e14762, 10.1111/iwj.14762.38356162 PMC10867290

The above article, published online on 14 February 2024, in Wiley Online Library (http://onlinelibrary.wiley.com/), has been retracted by agreement between the journal Editor in Chief, Professor Keith Harding; and John Wiley & Sons Ltd. Following an investigation by the publisher, all parties have concluded that this article was accepted solely on the basis of a compromised peer review process. The editors have therefore decided to retract the article. The authors did not respond to our notice regarding the retraction.

